# A synthetic biology approach for the treatment of pollutants with microalgae

**DOI:** 10.3389/fbioe.2024.1379301

**Published:** 2024-04-05

**Authors:** Luke J. Webster, Denys Villa-Gomez, Reuben Brown, William Clarke, Peer M. Schenk

**Affiliations:** ^1^ Australian Institute for Bioengineering and Nanotechnology, The University of Queensland, Brisbane, QLD, Australia; ^2^ School of Civil Engineering, The University of Queensland, Brisbane, QLD, Australia; ^3^ Algae Biotechnology Laboratory, School of Agriculture and Food Sustainability, The University of Queensland, Brisbane, QLD, Australia; ^4^ Algae Biotechnology, Sustainable Solutions Hub, Global Sustainable Solutions Pty Ltd, Brisbane, QLD, Australia

**Keywords:** microalgae, synthetic biology, genetic engineering tools, pollutants, plastics, salinity, pesticides, heavy metals

## Abstract

The increase in global population and industrial development has led to a significant release of organic and inorganic pollutants into water streams, threatening human health and ecosystems. Microalgae, encompassing eukaryotic protists and prokaryotic cyanobacteria, have emerged as a sustainable and cost-effective solution for removing these pollutants and mitigating carbon emissions. Various microalgae species, such as *C. vulgaris, P. tricornutum, N. oceanica, A. platensis,* and *C. reinhardtii,* have demonstrated their ability to eliminate heavy metals, salinity, plastics, and pesticides. Synthetic biology holds the potential to enhance microalgae-based technologies by broadening the scope of treatment targets and improving pollutant removal rates. This review provides an overview of the recent advances in the synthetic biology of microalgae, focusing on genetic engineering tools to facilitate the removal of inorganic (heavy metals and salinity) and organic (pesticides and plastics) compounds. The development of these tools is crucial for enhancing pollutant removal mechanisms through gene expression manipulation, DNA introduction into cells, and the generation of mutants with altered phenotypes. Additionally, the review discusses the principles of synthetic biology tools, emphasizing the significance of genetic engineering in targeting specific metabolic pathways and creating phenotypic changes. It also explores the use of precise engineering tools, such as CRISPR/Cas9 and TALENs, to adapt genetic engineering to various microalgae species. The review concludes that there is much potential for synthetic biology based approaches for pollutant removal using microalgae, but there is a need for expansion of the tools involved, including the development of universal cloning toolkits for the efficient and rapid assembly of mutants and transgenic expression strains, and the need for adaptation of genetic engineering tools to a wider range of microalgae species.

## Graphical Abstract

**Figure:**
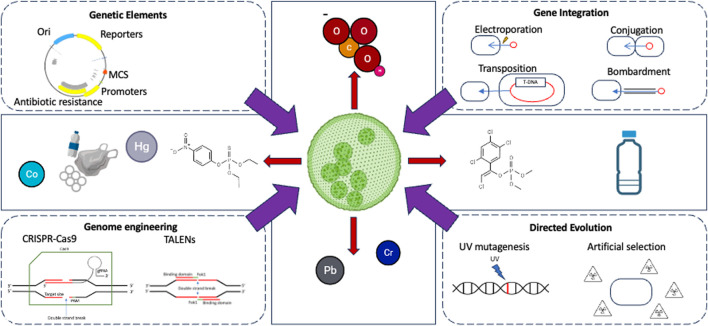
Created with Biorender.com.

## 1 Introduction

The increase in global population, combined with rapid development and industrial expansion have led to a significant number of pollutants released to water streams. This pollution originates from both domestic and industrial sources that release various organic and inorganic contaminants. Inaction to address water quality issues threatens human health, the economy and ecosystem health (UNEP, 2021). Metals are common in industrial/mining operations, and interactions with waterways can lead to severe environmental and health consequences (Tchounwou et al., 2012). Plastic pollution occurs from domestic and industrial sources with an estimated 9–23 million tonnes per year into aquatic environments, with harmful consequences on contaminated ecosystems (MacLeod et al., 2021). Pesticide pollutants arise mainly from agricultural operations, spreading to unintended soil, water and air systems, affecting non-target wildlife and human health (Verasoundarapandian et al., 2022). Highly saline wastewater is commonly generated industrially, and untreated release is known to adversely affect water ecosystems, agriculture, and reduce clean water availability (Lefebvre and Moletta, 2006).

Therefore, the development of technologies to remove pollutants has become a global target. Technologies based on physical and chemical methods are the most widely used in industry, yet these have significant drawbacks. Physical methods are often energy-intensive thereby indirectly generating greenhouse gas emissions if not powered by renewable energy. On the other hand, chemical methods require consumption of chemicals that may increase treatment costs and can potentially introduce new chemical by-products into the environment (Yao et al., 2012).

Biological methods, in contrast, can be more sustainable and cost-effective as they minimise the requirement for energy and reagents. The use of microalgae, including diatoms, green algae and cyanobacteria, has attracted attention for removing water pollutants due to their high versatility, rapid growth, ability to grow with limited resources, and the various high-value by-products (Borowitzka, 2013; Cui et al., 2021; Kalra et al., 2021). Additionally, the carbon sequestration ability of microalgae can help offset carbon emissions in parallel to cleaning the environment (Vale et al., 2020).

In the last decade, the application of synthetic biology tools to microalgae for biotechnological purposes, such as the production of biofuels and high-value molecules, has gained great interest. This has been accelerated due to the recent availability of genome sequences and other omics datasets from diverse microalgae species (Kumar et al., 2020). Genetic engineering of microalgae offers a unique advantage compared to conventional biological methods, which are constrained by the traits of the wild-type organism. With synthetic biology, these traits can be moulded to better fit the purpose of the pollutant removal strategy. Through this approach, synthetic biology and omics datasets are used to create engineered microalgal species with superior tolerance to toxic pollutants and higher uptake or transformation rate of pollutants as compared with microalgae wild type species. The approach of increasing tolerance, transormation and uptake rate of metals such as mercury (Hg), chromium (Cr), and arsenic (As) can also increase the range of applications (Ranjbar and Malcata, 2022). Furthermore, genetic engineering can create new pollution removal methods outside of the wild-type targets, such as creating PET plastic-eating microalgae as demonstrated by Kim et al. (Kim et al., 2020) and Moog et al. (Moog et al., 2019).

The progress made on genetically engineered microalgal species for pollutants removal is subject to the availability of genetic tools. This area has been left behind when compared to the increased progress carried out for model species such as *E. coli* and *S. cerevisiae* (Sproles et al., 2021). To address these needs, many research groups have been developing universal cloning toolkits for efficient and rapid assembly of transgenic expression strains, including promoters, terminators, reporters, selection markers, tags, untranslated regions (UTRs), and introns (Weber et al., 2011; Pollak et al., 2019; Valenzuela-Ortega and French, 2021). Toolkits like ULoop, have been designed to allow expression in all cellular kingdoms, yet most have been developed to focus on specific organisms. On the other hand toolkits for specific microalgal species are scarce, with only a few examples such as CyanoGate (Vasudevan et al., 2019) and the *C. reinhardtii* MoClo toolkit (Crozet et al., 2018). As seen in model species such as *E. coli*, access to standardised genetic tools can streamline research using microalgal strains for pollutants removal by allowing simple transfer between research groups, rapid mutant generation, and high throughput research.

There have been previous reviews centred around the premise of synthetic biology in microalgae in recent years. Most reviews in this field have focused on microalgal bioproduction, such as Naduthodi et al. (Naduthodi et al., 2021), Rock et al. (Rock et al., 2021), Ng et al. (Ng et al., 2020), and Cao et al. (Cao et al., 2023), which are centred around carbon metabolism, biofuel production, metabolic regulation, and natural pigment synthesis respectively. Fewer reviews have focused on synthetic biology for pollutant removal in microalgae, and these few have focused on wastewater bioremediation, such as the reviews by Sattayawat et al. (Sattayawat et al., 2021) and Hassanien et al. (Hassanien et al., 2023).

This review provides an overview of the advances of synthetic biology for pollutants removal, with a specific focus on the development of genetic engineering tools and how these have been and can be applied to microalgae as a workhorse for pollutants removal. This review first discusses the role of microalgae on pollutants removal and the major mechanisms behind bioremediation with a view on how these can be optimised using genetic engineering with the genetic engineering tools available. Finally, the review analyses how these tools have been utilised for bioremediation to date and point towards research gaps and future directions.

A narrative review approach was used to make the review, summarising the relevant literature and highlighting the significance of new research and gaps (Paré et al., 2017). Web of Science, PubMed, JSTOR, SCOPUS and Google Scholar databases were used to select the relevant studies studies in this review. Studies conducted in the last 5 years were prioritised, and publications prior to 2010 were excluded except for notably impactful research. Search terms including but not limited to “microalgae”, “bioremediation”, “synthetic biology”, “CRISPR”, “heavy metals”, “pollutant uptake” and “genetic engineering” were used and combined in an appropriate manner to identify the relevant research studies.

## 2 Microalgae-facilitated pollutant removal

### 2.1 Previous work on bioremediation using microalgae

Microalgae, including diatoms and green algae, together with cyanobacteria, consist of a large range of both eukaryotic protists and prokaryotic cyanobacteria, and therefore, there is significant genetic diversity within this definition, spanning two domains and the kingdoms of plantae, protista, and bacteria (Singh and Saxena, 2015). This broad range of organisms have demonstrated different capacities for pollutants removal (Supplementary Table S1). Most research on wild-type microalgae has involved *C. vulgaris* and *P. tricornitum*, while genetic engineering research has focused on the model microalgae *C. reinhardtii* or cyanobacteria such as *Synechococcus* sp,.

*C. vulgaris* is currently the most investigated eukaryotic microalgal species for wastewater treatment (Shetty et al., 2019). It is a robust species, with relatively high resistance to chemical/physiological changes (Wirth et al., 2020), which is partly why it shows great bioremediation potential. Li et al. (Li et al., 2021) cultivated *C. vulgaris* on membrane-treated distillery wastewater, and demonstrated removal efficiencies of 80, 94% and 72% of total nitrogen, phosphorous and chemical oxygen demand (COD), respectively, while simultaneously removing 99, 85% and 42% of calcium (Ca), magnesium (Mg), and molybdenum (Mo). *C. vulgaris* has demonstrated 96%–99% removal of common pesticides such as atrazine, carbofuran, dimethoate, and simazine (Hussein et al., 2017) as well as for heavy metals (Piccini et al., 2019; Goswami et al., 2022).

Another microalga of interest is *P. tricornutum*, a marine diatom that is well known for high accumulation of omega-3 fatty acids (up to 35% of cell weight) and enriched protein (Pudney et al., 2019). *P. tricornutum* has demonstrated rapid growth rates in saline wastewater and is of particular interest for remediation of both inorganic and organic contaminants in oilfield produced water where it has demonstrated 92, 76, 85, 72% and 56% removal of nitrate, phosphate, iron (Fe), fluorine (F), and Mg, respectively (Gillard et al., 2021). *P. tricornutum* is one of two algal species that have been investigated for transgenic biodegradation of polyethylene terephthalate (PET) plastic (Moog et al., 2019), as it has advanced genetic toolkits available (George et al., 2020).

*N. oceanica* is a green marine microalga with a small cell size (1–3 µm) and rapid growth rate. It has demonstrated large-scale waste bioremediation of industrial, municipal, and agricultural wastewater (Silkina et al., 2019), as well as removal of heavy metals, particularly lead (Pb) (Waluyo et al., 2020).

*A. platensis,* also known widely as spirulina, is a filamentous gram-negative cyanobacterium with photosynthetic capability. Unlike eukaryotic algae which can only consume inorganic carbon in the form of dissolved CO_2_, *A. platensis* can consume inorganic carbon in both the ionic and non-ionic forms of CO_2_ (HCO_3_^−^ and CO_3_^2-^). As such, spirulina has been of interest in the bioaccumulation of highly alkaline wastewater such as coal-seam gas water (Alsufyani, 2022).

*C. reinhardtii* is widely considered the model organism for green microalgae. As such, extensive genetic engineering research has been conducted in comparison to other algal species (Tran and Kaldenhoff, 2020). It is the second algal organism investigated for transgenic PET degradation (Kim et al., 2020). The species has been investigated for various heavy metal treatments, including As (Mohamed et al., 2022), manganese (Mn) and cadmium (Cd) (Ibuot et al., 2017), and even uranium (U) (Baselga-Cervera et al., 2018). Ibuot et al. (Ibuot et al., 2017) also noted, however, that despite the overexpression of *crMTP4*, increasing Cd tolerance and uptake compared to wild type, the genetically modified *C. reinhardtii* still showed lower levels of Cd tolerance and accumulation than naturally adapted chlorophyte algae strains. *C. reinhardtii* has also demonstrated remediation of chemical pollutants, such as potassium cyanide (Sobieh et al., 2022), phenols (Nazos et al., 2020) and fluroxypyr (Zhang et al., 2011).

Among the species with plastic removal/degradation capacity are *Anabaena spiroides*, *Scenedesmus dimorphus*, and *Navicula pupula*, isolated from dumped polyethylene (PE) waste bags. Kumar et al. (Kumar et al., 2017) determined that all three species proliferated at higher rates on low density polyethylene (LDPE) compared to high density polyethylene (HDPE), but the highest percentage of degradation was obtained from *A. spiroides* at 8.18%, followed by *N. pupula* and *S. dimorphus* at 4.44% and 3.74% respectively. Paladhi et al. (Paladhi et al., 2022) provide a full review of plastics degraded/absorbed by microalgae, although currently research remains limited, both in terms of plastic types and microalgal species investigated.

### 2.2 Mechanisms and biological targets

**FIGURE 1:**
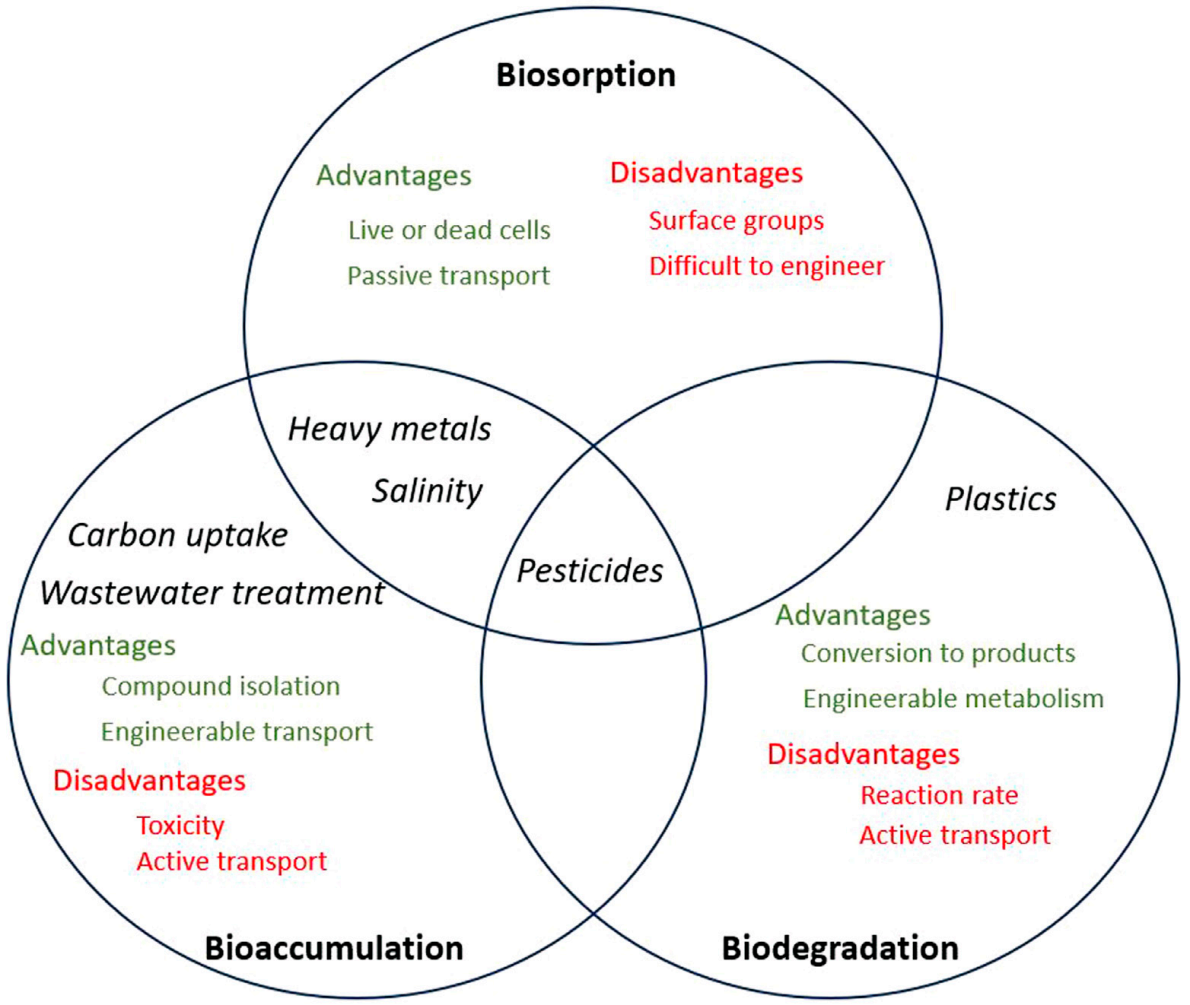
Venn diagram demonstrating biosorption, bioaccumulation and biodegradation. Black text indicates pollutant targets, green text indicates benefits of each method, while red text indicates drawbacks of each method.

The removal of pollutants using microalgal species involves biosorption, bioaccumulation, and biodegradation mechanisms. Figure 1 shows the mechanisms involved in the removal of the targeted pollutants discussed in this review as well as the advantages and disadvantages of each method, which will be discussed more in detail in the following sections.

Biosorption is a mass transfer process by which the pollutant is displaced from the liquid phase and chemically bound to the cell (Abdelfattah et al., 2023). Bioaccumulation is a much slower metabolic process requiring cellular energy, whereby the cell actively takes up a substance and metabolises or accumulates it (Mustafa et al., 2021). Biodegradation involves the use of cellular enzymatic reactions to break down target pollutants into safe, simple chemicals, such as CO_2_, H_2_O, ethylene glycol, etc. This can occur both intracellularly or extracellularly, depending on reaction pathways and enzyme localisation (Hong et al., 2013).

#### 2.2.1 Microalgae for heavy metal removal

Heavy metals are naturally occurring elements found throughout the Earth’s crust, yet significant increase in mining operations and industrial use has caused higher than tolerable concentrations in soil and water (Tchounwou et al., 2012). Many microalgae uptake heavy metals such as cobalt (Co), copper (Cu), Fe, Mo, Mn, and zinc (Zn) for cell metabolism, but heavy metals such as As, Cd, Cr, Pb and Hg are typically toxic for many strains. However, microalgae strains hold varied tolerance and bioaccumulation capability towards heavy metals.

In addition, different functional groups as well as proteins and peptides are responsible for metal-binding, which can be used for heavy metals removal (Leong and Chang, 2020). Chelating reactions are a key component for detoxification of metals in organic matter. Phytochelatins and phytochelatin synthases are essential players in the bioremediation process for plant or algae metal processing (Balzano et al., 2020; Uraguchi et al., 2022). These chelatins serve as small metal-binding ligand peptides. As heavy metal toxicity is generally caused by ion exchange - where the new metal replaces the position of essential metal cofactors in enzymes, the binding of the metals to ligand peptides blocks the ion exchange process and thus increases metallic resistance and uptake. Microalgae specifically, are able to utilise ion exchange with cell surface cations, such as Ca, sodium (Na), and Mg, to sequester heavy metals present in the medium. These metals can then be extracted via algal harvesting. However, sequestration ability is highly dependent on metal toxicity, if using live cell mass (Leong and Chang, 2020).

A common method to increase the effectiveness of heavy metal biosorption in microalgae is to utilise immobilised microalgae biomass on the surface of Na-alginate (or other biopolymer) beads (Rinanti et al., 2022). The immobilised biomass has the advantage of being easier to process, increased surface area, and can be regenerated. This technique has been utilised with biomass from *Chlorella* sp. (Siwi et al., 2018; Wilan et al., 2019; Wilan et al., 2020; Rinanti et al., 2022), *Desmodesmus* sp. (Widyaningrum et al., 2021), *Scenedesmus obliquus* (Wilan et al., 2019; Wilan et al., 2020; Rinanti et al., 2022), and *Calothrix* sp. (Ameri et al., 2019). Notably, a transgenic strain of *C. reinhardtii* was previously immobilised on microalgal beads and demonstrated a significant increase in metal biosorption when compared to the beads composed of wild-type cells (Ibuot et al., 2020), indicating that there is potential for synthetic biology based improvement of this method.

Due to the wide range of detoxification mechanisms, there are many possible gene targets for heavy metal remediation in microalgae. For example, chelatin synthase genes are highly promising, due to their ability to activate phytochelatins accumulation with exposure to metal ions, and drive sequestration of the ions in the vacuole (Li et al., 2020). Cell wall protein-encoding genes also serve as a good target and provide the ability to engineer the cell surface ligands available. Additionally, metal transporter genes, which encode proteins/peptides that facilitate transfer intracellularly, offer interesting gene targets, yet heavy metals are typically far more toxic in the cell cytosol than when bound to the cell surface (Nowicka, 2022). This may be a limiting factor for this method. Piccini et al. (Piccini et al., 2019) investigated the use of *C. vulgaris* and *A. platensis* for bioremediation for Nickel (Ni), Zn, Cd, and Cu, while simultaneously producing bio-crude oil. They demonstrated 80% and 99.5% recovery by biosorption from *C. vulgaris* and *A. platensis,* respectively, across all tested metallic species in a 10 mM solution.

#### 2.2.2 Microalgae for salinity removal

Based on the rapid expansion of industrial processes in recent years, a low-energy desalination technology has become increasingly sought after. Industrial processes such as coal-seam-gas fracking, tannery operation, and mining, all produce highly saline wastewater, often rich in carbonates that cannot be safely released to the environment without risk (Millar et al., 2016). Microalgal species have demonstrated a high affinity for salt remediation from these wastewater sources, without the need for significant energy or chemical input while simultaneously generating valuable products such as omega-3 oil, phycocyanins, dyes, carotenoids, and biodiesel (Gan et al., 2016). Salinity removal through microalgae can occur through biosorption (internalisation of salt ions, also known as bioabsorption) and bioadsorption (binding of salt ions to the surface of the cell wall) given the negative charge of their cell wall (Wei et al., 2020). Due to the net negative charge of the algal cell wall, algal biomass has a higher affinity for cationic ion removal, such as Na and Mg.

In addition, certain microalgae such as cyanobacteria can utilise carbonate salts as a carbon source and this bioaccumulation process can be used for inorganic carbon removal (Muñoz-Marín et al., 2020). Unlike traditional carbon fixation processes such as the Calvin-Benson cycle, which utilises CO_2_ as the starting point, cyanobacteria can utilise HCO_3_^−^ as the active species either through the 3-hydroxypropionate, 3-hydroxypropionate-4-hydroxybutyrate, and dicarboxylate-4-hydroxybutyrate cycles (Wang et al., 2015), or through the use of a modified Calvin-Benson cycle, with the use of bicarbonate ion transporters and carbonic anhydrase enzyme (Badger and Price, 2003).

#### 2.2.3 Microalgae for pesticides removal

Chemical pesticides are widely used compounds that significantly reduce crop loss and enhance agricultural productivity (Verasoundarapandian et al., 2022), yet persistent pesticides can cause large-scale ecological damage when used consistently. Pesticides are typically classified by target species and chemical structure, which are highly variable and therefore, challenging to be removed from the environment (Varanasi et al., 2016; Venegas-Rioseco et al., 2021). Removal of pesticides by microalgae is based on biosorption, bioaccumulation and biodegradation and its effectiveness is highly dependent on interactions in the cell wall, lipid content, and metabolic pathways, which vary among species (Wang et al., 2019). Biosorption is a fast and energetically passive mechanism that involves the binding of pesticide species to functional groups on the surface of the cell wall can be one either with dead or live algal cells (Goher et al., 2016). On the other hand, bioaccumulation involves the uptake of the target pesticides into the cell through ion transfer mechanisms. Biodegradation is an extension of bioaccumulation. After the toxic compounds are accumulated, some species can degrade the compound into non-toxic metabolites, which can help to supplement metabolic activity and cell growth. Biodegradation effectiveness relies on the presence of specific enzymes such as hydrolases, esterases and transferases.

#### 2.2.4 Microalgae for plastics removal

Over the last century, plastics have become increasingly prevalent and essential to modern life. The majority of waste plastic is not bio-degradable and is able to persist in the environment for centuries (MacLeod et al., 2021). Some plastics have been demonstrated to biodegrade in the presence of microalgal species. For example, Sanniyasi et al. (Sanniyasi et al., 2021) isolated an algal species *Uronema africanum* from a waste plastic bag. They demonstrated that the algae could colonise the surface of a LDPE sheet and initiate degradation within 30 days. However, most of the research on plastic degradation in recent years has focused on the recently discovered PETase, isolated from *Ideonella sakaiensis*. This enzyme is a cutinase-like serine hydrolase that effectively removes one monomer of MHET from the PET chain in each reaction cycle (Jerves et al., 2021). A second enzyme, MHETase, is then able to break down the MHET monomers into terephthalate and ethylene glycol, which are much simpler carbohydrates that can be used as energy and carbon stores for the cell. The enzymatic structure and mechanisms of MHETase is not as well understood as PETase, but it has been shown that an artificially linked chimeric form of PETase and MHETase shows greater enzymatic activity overall when compared to the activity of a mixture of the two free enzymes (Knott et al., 2020). While algae cannot directly remediate PET, there is potential to harness the genetic power of *I. sakaiensis*, incorporating the genes for PETase and MHETase to transport these mechanisms into algal cells.

## 3 Genetic engineering tools for synthetic biology

### 3.1 Principles

**FIGURE 2:**
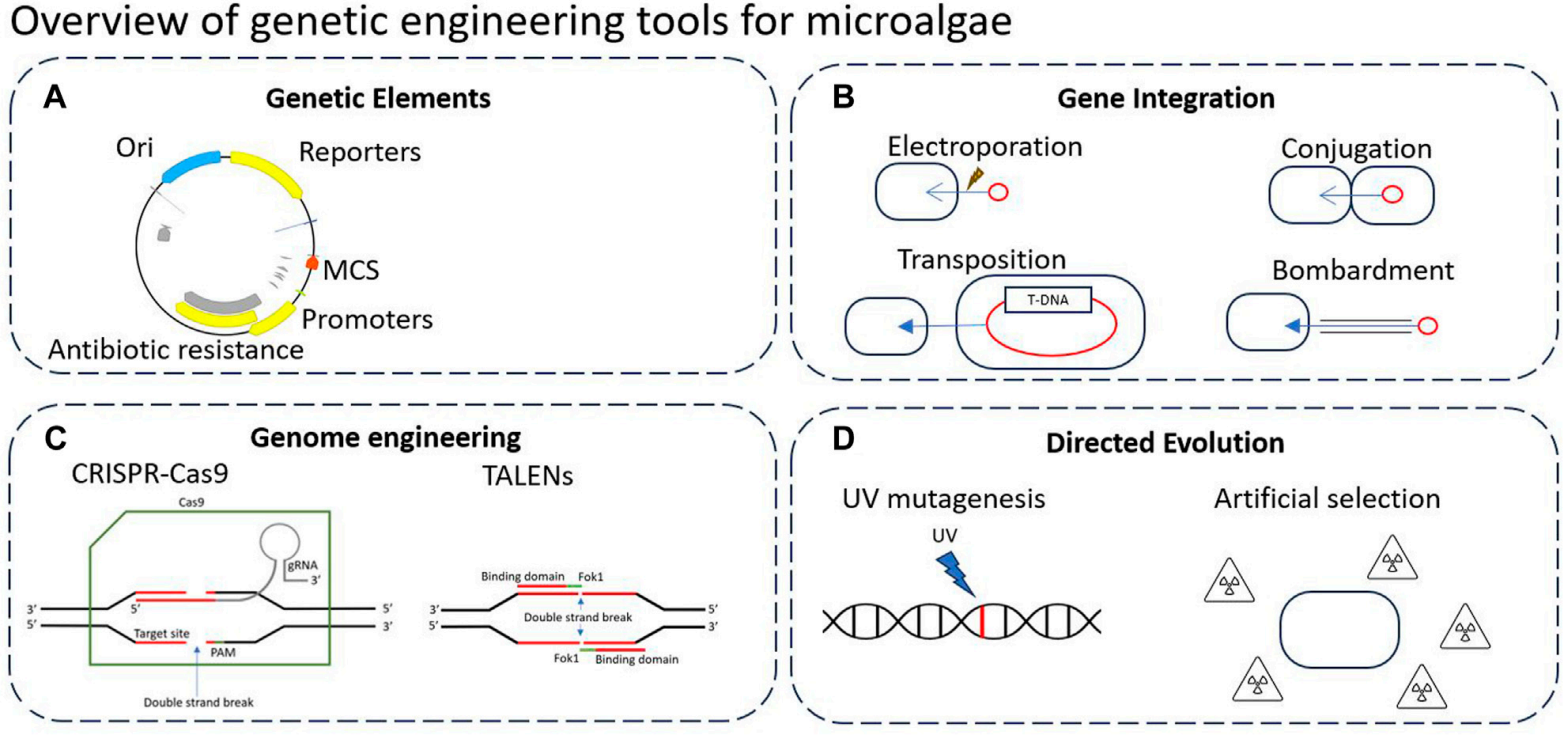
Overview of genetic engineering tools that can be applied to microalgae. **(A)** Typical genetic elements in plasmid design. **(B)** Typical methods of gene integration. **(C)** Precise genome engineering tools such as CRISPR/Cas9 and TALENs. **(D)** Typical directed evolution approaches to modify DNA.

Figure 2 below highlights the key genetic engineering tools that can be applied to microalgae. Genetic elements design is generally crucial to all genome engineering technologies, yet there is currently no universal standardised assembly system which makes building from previous work difficult (Figure 2A). For this, there is a wide variety of genetic elements used, including promoters, terminators, multiple cloning sites (MCSs), origins of replication/transfer, resistance genes, plasmid backbones, transposons and reporters (Nora et al., 2019).

An important step of genetic engineering is gene integration (Figure 2B), which is the insertion of DNA into the genome of the host cell or maintained as a plasmid/episome. Gene integration follows a variety of methods such as conjugation, electroporation, biolistic bombardment, or transposition (discussed below).

Recently, there has been a large range of precise genome engineering tools developed such as CRISPR/Cas9 and TALENs, which are often assembled *in vivo* using genetic elements carried by a plasmid (Figure 2C). These tools allow for precise and targeted editing of the genome, as opposed to many previous methods which rely upon recombination events to occur (Liang and Yu, 2021).

Finally, directed evolution refers to the DNA modification without utilising genetic elements or introducing foreign DNA (Figure 2D). Directed evolution aims to mimic and expedite natural evolution processes, typically by introducing stronger selection forces or mutagenic pressure. Through iterative rounds of gene diversification and screening, these techniques can be used to rapidly generate novel mutants with altered phenotypes (Wang et al., 2021). While this review is focused on synthetic biology based genetic engineering, Zhang et al. (Zhang et al., 2021) provide a review describing directed evolution and adaptive laboratory evolution (ALE) techniques and research in microalgae. Directed evolution, as well as other non-GM techniques for enhanced biomass and metabolite yields in microalgae is further reviewed by Wass et al. (Wass et al., 2021).

### 3.2 Genetic elements and assembly

Genetic element design remains the first crucial step for synthetic biology research. Regardless of the desired modification, a plasmid-based system remains the most common method of introducing DNA into the host cell. The generation of a plasmid for DNA delivery has a large variety of techniques available. A plasmid used for DNA delivery in a eukaryotic organism typically contains the following genetic elements: a) Bacterial resistance marker such as ampicillin (for cloning in propagation organism, e.g., *E. coli*), b) Eukaryotic resistance marker such as hygromycin (for selection in target organism), c) Gene cassette for gene of interest (in a CRISPR system this may include a promotor, the *Cas9* gene, an encoded single guide RNA and a terminator), d) an origin of transfer, e) an origin of replication (both bacterial and eukaryotic), and, f) a reporter gene, such as *GFP* (Sinah et al., 2012). Prokaryotic plasmids are typically much simpler and lack the eukaryote-specific elements previously mentioned.

**FIGURE 3:**
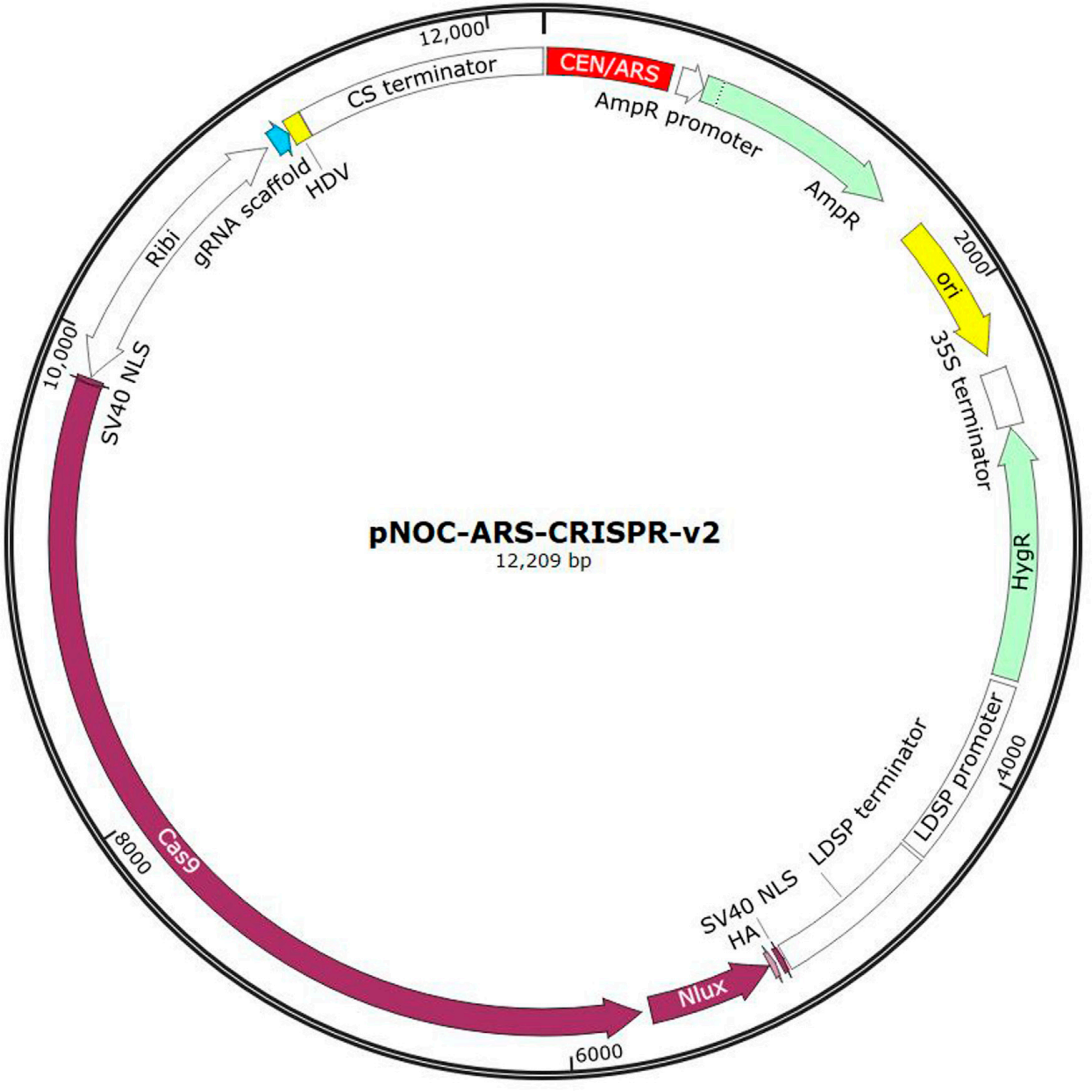
Example of the pNOC-ARS-CRISPR-V2 CRISPR delivery plasmid developed for N. oceanica (Image generated from SnapGene).

An example of a plasmid used for DNA delivery is shown in Figure 3, which shows a CRISPR delivery plasmid developed for *N. oceanica*. This system contains a bidirectional promotor (Ribi) which encodes for the Cas9 protein as well as the associated single guide RNA (sgRNA). The Cas9 protein is tagged with Nlux, a luciferase reporter tag. Ampicillin and hygromycin resistance genes are included for bacterial and eukaryotic expression respectively. A CEN/ARS sequence is integrated for autonomous replication in the eukaryotic cell. A bacterial origin of replication is also included (ori) (Poliner et al., 2018).

Typically, assembly of plasmids requires a series of molecular biology techniques, including standard systems such as PCR and restriction digestion, but may include more modern approaches such as Gateway cloning, Gibson assembly, and Golden Gate cloning.

Golden Gate cloning is fast growing in popularity due to its ease of assembly, standardisation, and ability to incorporate multiple levels of assemblies (Marillonnet and Grützner, 2020). While typical cloning relies upon type II restriction enzymes which cut within the recognition site, Golden Gate cloning utilises type IIs enzymes which cut the sequence a few bases downstream of the binding site. This specialisation allows for insertion of DNA without removing the enzyme recognition sequence and allowing for seamless, scar-free cloning. A secondary type IIs restriction enzyme site can be incorporated for higher level cloning. Golden Gate cloning also has the advantage of modularity and consistency of cloning. This cloning mechanism uses the standardised MoClo format, defines DNA parts as building blocks upon which to build larger genetic circuits. This system utilises standard overhangs for assembly of transcriptional units under a common syntax (Weber et al., 2011; Patron et al., 2015). For example, a MoClo compatible promotor part (AB) would have the 5′ overhang of GGAG and a 3’ overhang of TACT. This allows for parts to be reused between researchers without the need for labour intensive design and cloning steps.

### 3.3 Gene integration techniques

#### 3.3.1 Conjugation

Conjugation is the transfer of plasmid DNA from a bacterium to another organism. Conjugation has been observed to be a universally conserved DNA transfer mechanism among bacteria, and conjugative elements typically contain all of the genes required for both vertical and horizontal DNA transfer (Virolle et al., 2020).

While the receiver organism is typically another bacterium, this can be harnessed to transfer plasmids into microalgae. This technique is commonly used for cyanobacteria, but its effectiveness for eukaryotic algae is limited by the recipient strain’s ability to receive and maintain the foreign DNA (Gutiérrez and Lauersen, 2021). Conjugation methods have been developed for many microalgae, such as *Acutodesmus obliquss* and *Neochloris oleoabundans* (Muñoz et al., 2019), and *Phaeodactylum tricornutum* (Sharma et al., 2018). Random incorporation of the DNA can cause random knock-out mutants, which may be undesirable. As such, conjugation may be combined with specific chromosomal integration systems like CRISPR, transposons, or is expressed as episomes.

#### 3.3.2 Electroporation

Electroporation is the process by which DNA can be taken up into a host cell by generating temporary pores in the cell membrane through the application of electric current. In microalgae, electroporation was first optimised in *C. reinhardtii* (Shimogawara et al., 1998), and this technique increased the transformation efficiency compared to previous methods 100-fold. Since then, electroporation methods have been described for many species including *N. oceanica* (Kilian et al., 2011) and *P. tricornutum* (Niu et al., 2012). However, the main hurdle to overcome for many microalgae is the low transformation efficiency. Poveda-Huertes et al. (Poveda-Huertes et al., 2023), investigated novel transformation strategies to improve the efficiency of electroporation in *N. oceanica* and *P. tricornutum*. They found that transformation of *N. oceanica* cells during the G2/M phase increased transformation efficiency 8-fold, and when incorporated with saponin as a transformation adjuvant, efficiency was increased >10-fold. Saponin was hypothesised to increase the cell wall permeability, thereby increasing DNA intracellular transfer. In *P. tricornutum*, saponin as a transformation adjuvant doubled transformation efficiency. Similar to conjugation, integration of the DNA can cause random knock-out mutants and may therefore be combined with other strategies.

#### 3.3.3 Bombardment

Biolistic bombardment is a common method used to insert DNA into cells, as it can be used to target nuclear, mitochondrial, or chloroplast genomes (Gutiérrez and Lauersen, 2021). The method involves DNA-coated metal particles being fired from a “gene gun”. This method can be limited by the cells’ ability to recover from bombardment, and often has a lower transformation efficiency. This method has been utilised in many microalgae, including *Haematococcus pluvialus* (Yuan et al., 2019), *P. tricornutum* (Apt et al., 1996), and the chloroplasts of *C. reinhardtii* (Ramesh et al., 2011). It is one of few methods that can specifically target chloroplast or mitochondrial DNA.

#### 3.3.4 Episomal DNA expression

Another way to manipulate gene expression within a cell is episomal expression. An episome is a system of DNA that can replicate autonomously without being integrated into genomic DNA (Tanwar and Kumar, 2020). This approach does not modify nuclear DNA, but generally instead exists as a plasmid. Autonomous replication can be facilitated by the inclusion of an autonomously replicating sequence (ARS), typically obtained from yeast (Kurita et al., 2022). Additionally, a selection marker is required to maintain the presence of the plasmid between generations. Episomal expression may be preferred as a preliminary technique to express a gene of interest for genetic studies where chromosomal integration would be too difficult or time consuming. Episomal systems can also be a carrier for gene modification proteins such as Cas9, such as the previously mentioned Poliner et al. (Poliner et al., 2018) study in microalga *N. oceanica*. Through the episomal Cas9 system, knock-out algae mutants can be obtained without the introduction of any foreign DNA once the episome is cured.

#### 3.3.5 Transposons

Transposable elements (TE), also known as transposons, are DNA sequences that can migrate between genomic locations. Eukaryotic genomes contain a multitude of TE and take up a large portion of the genome (Munoz-Lopez and Garcia-Perez, 2010). TE have played a critical role in evolution due to their ability to transport DNA, and if harnessed, can be utilised to integrate genes of interest into the host genome (Ivics and Izsvák, 2010). Transposons are typically comprised of a transposase gene surrounded by terminal inverted repeats (TIR). The transposase recognises the TIR sites and excises the gene from its original location and transports it to a new site. This can be harnessed to cause reversible loss-of-function mutations in affected genes. The process by which this occurs varies among the different TE families.

TE can also be used to transfer genes of interest into host cells. A donor plasmid, containing the gene of interest flanked by TIR’s will be recognised by transposase and can be transferred from the plasmid into the cell’s chromosomes (Mátés et al., 2009). Of discovered transposons, *Sleeping Beauty* (SB) is one of the most widely used genetic tools. Cre/*loxP* is another transposition system, utilising site-specific recombination to delete or insert DNA at target sites (Sengupta et al., 2017). The Cre/*loxP* system has demonstrated use in microalgal systems. Kasai and Harayama (Kasai and Harayama, 2016) utilised this system to generate marker-free transgenic strains in *C. reinhardtii*. By inserting *LoxP* recognition sites flanking the reporter gene, they were able to then remove this sequence by expressing a synthetic Cre recombinase, without impacting the transgene expression cassette. This study presents an interesting technique to generate marker-free transformants in microalgae, an integral step for the integration of genetically engineered microalgae for industrial use.

Another recombination system is the Flp-*FRT* derived from the yeast *S. cerevisiae*. The Flp protein, colloquially known as flippase, catalyses the recombination of sequences between short flippase recognition target (*FLP*) sites, which are approximately 34 bases long. It has been used as a method to generate markerless transformants by excising the genetic marker after screening has occurred (Ji et al., 2017). Tan et al. (Tan et al., 2013) demonstrated this purpose in both *Synechocystis* spp. and *S. elongatus* by transforming a plasmid encoding the flippase gene, along with a kanamycin resistance gene flanked by the *FLP* sites and determined that the resistance marker was excised from the genome under conditional flippase expression, thereby generating marker-free transformants.

Osorio et al. (Osorio et al., 2019) utilised the transposome complex Tn5 to generate random mutations in *N. oceanica*. The high efficiency of transformation driven by a CMV viral promotor allowed for rapid generation of mutants and selection driven by the Tn5 encoded antibiotic-resistance cassette. High-throughput screening allowed for the identification of desired altered phenotypes–in this case, changes to accumulation of intracellular lipids. This allowed for identification of new genes involved in lipid metabolism.

#### 3.3.6 Agrobacterium tumefaciens

*Agrobacterium tumefaciens,* a plant pathogen, has been widely utilised to genetically engineer plant genomes due to its ability to transfer T-DNA from Ti plasmids. T-DNA is defined as the DNA region exchanged from *Agrobacteria* to the plant cells (Fortin et al., 1993), acting as a transposition system. These regions are flanked by conserved recognition sites approximately 25 bases in length (Jouanin et al., 1989), which are targets of the bacterial endonucleases, as well as several integral proteins, which excise the DNA region from the plasmid (Gelvin, 2003). These T-borders can be utilised for genetic engineering and insertion of desired DNA. However, its use is limited to plant, algal and fungal cells.

By utilising a modified *Agrobacterium* strain, Pratheesh et al. (Pratheesh et al., 2014) developed an efficient and improved method of DNA transformation to the microalga *C. reinhardtii*. Flanking the desired DNA insertion with the left and right T-border DNA regions allows for transformation into the host cell in the desired orientation via a binary plasmid system. However, this system does not allow for specific genomic location targeting, and as such, DNA may be inserted into transcriptionally silent genomic regions (Day et al., 2000). To combat this, reporter genes such as *GFP* are typically inserted along with genes of interest, and colonies screened by level of expression (Dong and Ronald, 2021).

Zhang et al. (Zhang et al., 2018) also utilised *Agrobacterium* transformation techniques to insert a CRISPR/Cas9 system in wheat. Other notable systems of Cas9 gene integration in wheat species typically results in multiple insertions, yielding high levels of the Cas9 protein, significantly increasing off-target mutations. By using the *Agrobacterium* method, off-target mutations were reduced while maintaining a high level of mutation efficiency (54.17%). This *Agrobacterium/*Cas9 system could be further applied to microalgae as the *Agrobacterium* method offers 20%–90% mutation efficiency compared to other methods such as 5%–12% for biolistic-mediated transformation based on evidence in plants (Nymark et al., 2016).

### 3.4 Precise genome engineering

#### 3.4.1 CRISPR/Cas9

CRISPR (clustered regularly interspaced short palindromic repeats) has stood in recent years as one of the easiest and most efficient gene modification tools (Fu et al., 2019). CRISPR DNA sequences were isolated from bacterial organisms where they were typically used as antiviral defence mechanisms. CRISPR sequences target specific DNA and were thus used by the antiviral machinery to detect viral nucleic acids and aid in its breakdown. In combination with the Cas9 enzyme, which cleaves specific DNA structures based on its paired CRISPR sequence, the enzyme/RNA complex can be utilised to insert a double-strand-break in targeted DNA (Jiang and Doudna, 2017). The double-strand-break can then be used for a variety of purposes (Adli, 2018). A simplified diagram of the CRISPR/Cas9 mechanism of action is shown in Figure 2C, where the guide RNA is shown to direct the Cas9 nuclease to cut upstream of the protospacer adjacent motif (PAM) sequence.

#### 3.4.2 CRISPR/Cas9 for gene inactivation/knockout

Upon the creation of a double strand break inside the intron of a gene, the host cell machinery will attempt to repair the damaged DNA. The most predominant repair mechanism is non-homologous end joining (NHEJ). This has the possibility to introduce indel mutations (an insertion of nucleotide at the target site) which can lead to frameshift mutants (Chang et al., 2017). A frameshifted gene will be unable to produce the associated protein, thus creating a “knockout” mutant (a strain modified to be unable to produce a targeted protein).

U6 promoters have typically been used to drive high expression of sgRNA sequences for CRISPR/Cas9 systems. U6 promoters are class III RNA polymerase III promoters that drive high expression and facilitate higher CRISPR efficiency, especially when endogenous U6 promoters are used (Long et al., 2018). However, many species, including oleaginous microalgae, have limited endogenous non-mRNA polymerase III promoters identified for this purpose (Kurita et al., 2023).

Other systems for transcription of sgRNA then need to be used. Poliner et al. (Poliner et al., 2018) utilised an episomal CRISPR/Cas9 system to generate marker-free gene knockouts in *N. oceanica*. The system used sgRNA flanked by the hammerhead and hepatitis delta virus self-cleaving ribozymes to create precise 5′ and 3’ ends, which helped to overcome the low KO efficiency (1%–4%) associated with previous CRISPR work in microalgae (Wang et al., 2016). The study aimed at knocking out nitrate reductase, which is an efficient marker for gene disruption, as KO strains then require supplementation with ammonia salts, as opposed to the regular nitrate media.

#### 3.4.3 CRISPR/Cas9 for gene insertion (knock-in)

Host repair mechanisms can also be utilised to insert DNA using CRISPR, as opposed to introducing indel mutations. Double strand break can be repaired by homologous recombination (HR), which utilises regions of homology to repair the damage. This process is longer, but less error prone than NHEJ. Homology regions can stem from sister chromatids; however, this process can also be harnessed for DNA manipulation using the CRISPR/Cas9 technology. This strategy utilises short homology arms (1–2 kb) flanking the integration cassette (DNA to be inserted), which serves as template DNA for the host repair mechanism (Kanca et al., 2019). Using the new template DNA, the HR repair mechanism is able to insert the integration cassette. This can be used to insert genes into the host genome, tag genes with reporters, or in some cases, insert whole pathways. However, the efficiency of HR-based CRISPR is reduced with larger inserts, which can be a major issue considering that NHEJ repair already dominates the repair mechanism in the cell population, leading to large screening requirements (Zhang et al., 2015).

Knock-in studies using CRISPR/Cas9 in microalgae have been limited so far, and studies have mainly focused on cyanobacteria due to the simplicity of the genome compared to eukaryotic algae (Naduthodi et al., 2018). The toxicity of Cas9 to algal cells, combined with the low efficiency of HR compared to NHEJ, has been the primary limitation for these studies. Ungerer and Pakrasi (Ungerer and Pakrasi, 2016), used a CRISPR system to insert the fluorescent *eYFP* reporter gene into the cyanobacterium *Synechococcus* 2,973, and demonstrated a 20% knock-in efficiency for transformed cells.

#### 3.4.4 CRISPR/Cas9 alternative technologies

Alternative forms of the CRISPR system are available, which can be used in conjunction with other technologies to enhance possible genome development strategies. dCas9 is one such example, which is a catalytically inactivated Cas9 protein that can be used to target gene locations without inducing strand breaks (Duke et al., 2020). It can also be bound to other protein systems to induce epigenetic changes. Various Cas9 orthologues such as Cas12a have been discovered/engineered targeting different PAM sequences such as NG, GAT, etc., which can improve specificity or gene localisation for certain applications (Paul and Montoya, 2020). Additionally, Prime Editing has recently been of interest for its potential to carry out precise nucleotide substitutions (Anzalone et al., 2019) without creating double-strand-breaks that can be lethal for many cells and lead to low HDR efficiency (Abdullah et al., 2020).

TALEN (transcription activator-like effector nucleases) were isolated from *Xanthomonas* proteobacteria. Naturally this bacterium injects a highly conserved repeat sequence into plant cells to alter transcription and allow for bacterial colonisation (Joung and Sander, 2013). This repeat domain serves as a recognition sequence for targeted DNA binding. This sequence structure can be attached to a nuclease (typically Fok1 endonuclease) to create a double-strand-break at the targeted sequence. While CRISPR is generally considered to be the most useful genetic engineering technique, TALEN do not require specific PAM sequences to generate double-strand-break and are the most precise tool based on percentage of off-site activity (Bhardwaj and Nain, 2021).

Similar to Poliner et al. (Poliner et al., 2018), Kurita et al. (Kurita et al., 2020) instead used TALENs to knock-out the nitrate reductase gene, as well as an acyltransferase in *N. oceanica*. The study highlighted the specificity of TALENs with no detectible off-target mutations while maintaining 56%, 31%, and 19% mutation efficiency for nitrate reductase, acyltransferase, and double mutants, respectively.

## 4 Applications of genetic engineering for pollutant removal

The previous sections reviewed the key genetic engineering tools that can be used for microalgae. In this section, we touch upon the tools that have been directly applied for pollutants removal. So far, these tools have targeted various heavy metals, salinity, pesticides, and recently, PET plastic (Table 1). Among algal species, *C. reinhardtii* has been the most common species for pollutants removal, yet there is large potential for *P. tricornutum*, *C. vulgaris*, and *S. elongatus* to also be used as workhorses for this purpose. Species selection has been largely dependent on available technologies, as well as species growth conditions and limitations. More engineering tools have been developed for the model algal organism *C. reinhardtii* (Crozet et al., 2018), which may explain its predominant use over other organisms.

**TABLE 1:** Various important examples of genetically engineered microalgae for bioremediation purposes.

Species	Tools used	Gene target(s)	Target pollutant	Reference
*Synechococcus elongatus*	Plasmid Conjugation	CPA1/2/3	Various Salts	Cui et al. (2020)
*Synechococcus elongatus*	Plasmid Transformation	*katmr*	Malachite green dye	Han et al. (2020)
*Chlorella vulgaris*	Plasmid Transformation	*rbcS, aldolase*	CO_2_	Yang et al. (2017)
*Chlamydomonas reinhardtii*	Biolistic Transformation/*Agrobacterium* transformation	*CrMTP4/AtHMA4*	Heavy metals (Cd, Mn/Cd, Zn)	Ibuot et al. (2017), Ibuot et al. (2020)
*Chlamydomonas reinhardtii*	*Agrobacterium* transformation	*GST*	Pesticides	Ismaiel et al. (2019)
*Chlamydomonas reinhardtii*	Plasmid electroporation	*LCI1*	Inorganic carbon	Ohnishi et al. (2010)
*Chlamydomonas reinhardtii*	Plasmid electroporation	*IsPETase*	PET Plastic	Kim et al. (2020)
*Chlamydomonas reinhardtii*	*Agrobacterium* transformation	*CYN*	Cyanide	Sobieh et al. (2022)
*Phaeodactylum tricornutum*	Biolistic Transformation	*IsPETase*	PET Plastic	Moog et al. (2019)
*Anabaena* sp	Plasmid electroporation	*linA2*	Lindane pesticide	Chaurasia et al. (2013)

### 4.1 Genetically engineered microalgae for heavy metal removal

Ibuot et al. (Ibuot et al., 2017) utilised biolistic bombardment to localise a plasmid carrying the metal tolerance protein (CrMTP4) into the nuclear genome of *C. reinhardtii*. Overexpression of this endogenous gene increased the Mn uptake of the alga by up to 2-3-fold compared to wild-type, yet there was no increase in Mn tolerance. However, Cd tolerance was increased, allowing the mutant strains to survive in 0.5 mM Cd supplemented media. However, when compared to different microalgae (*Chlorella luteoviridis*, *Paraclhlorella hussii* and *Parachlorella kessleri*) species isolated in Cd contaminated areas, Cd tolerance and uptake was significantly lower in the mutant strain, indicating that natural adaptation (likely multigenic and epigenetic) contributes more than single gene overexpression.

Ibuot et al. (Ibuot et al., 2020) further expanded metal tolerance in *C. reinhardtii* by transgenic expression of a plant Cd and Zn transporter (AtHMA4), both as a full-length protein and as a C-terminal tail which was known to contain the metal binding sites. They discovered similar increases in Cd and Zn tolerance for both the full-length protein and terminal domain, indicating that the increase was mainly due to the terminal binding region rather than metal transport.

Sattayawat et al. (Sattayawat et al., 2021) developed a narrative synthetic biology workflow to guide development in microalgal metal remediation and identified 49 unique genes/proteins based on the heavy metals Mn, Fe, Cu, Zn, As, Cd, Hg and Pb. However, many microalgal metal transporters still remain to be identified at a molecular level (Ranjbar and Malcata, 2022), and proteomics/transcriptomics offers an approach to identify such proteins for further expansion of genetically engineered metal remediating strains of microalgae.

### 4.2 Genetically engineered microalgae for salinity removal

Cui et al. (Cui et al., 2020) focused their research on the cyanobacterium *S*. *elongatus*, with the goal of developing the species as a photosynthetic microbial factory. However, large scale utilisation of this species requires large quantities of seawater, yet *S. elongatus* is sensitive to salt stress. The research team overexpressed 21 exogenous ion transporters, and identified three Mrp antiporters that, when overexpressed, increased cellular growth by up to 57.7% under high salt conditions. This research demonstrates that genetic engineering can be applied not just to increase uptake, but also to increase the range of systems for cultivation. For example, this species could be utilised in bioremediation strategies of high-salt wastewater from mining/industrial processes, reducing the waste exposed to the environment.

### 4.3 Genetically engineered microalgae for pesticides removal

Genetically engineered microalgae have shown potential for treatment of many chemical pollutants, including chemical dyes, pesticides, and even the highly toxic cyanide. Again, *C. reinhardtii* has seen the most usage for these studies. Sobieh et al. (Sobieh et al., 2022) utilised *Agrobacterium* to incorporate the cyanobacterial cyanase gene into *C. reinhardtii*. The transgenic strain demonstrated significant increases in resistance to cyanide toxicity and the ability to phytoremediate up to 150 mg/L of potassium cyanide. Sobieh et al. (Sobieh et al., 2022) propose that the engineered strain could be an effective treatment system for industrial cyanide containing wastewater. Ismaiel et al. (Ismaiel et al., 2019) utilised *C. reinhardtii* as a chassis for the glutathione-S-transferase enzyme using *Agrobacterium-*mediated transformation. This enzyme is responsible for degradation of many herbicides, and as such, the transgenic *C. reinhardtii* was demonstrated to reach up to 93.6% removal of the rice herbicide penoxsulam compared to the wild type at 52%.

Biodegradation of lindane, a common agricultural insecticide, has been conducted in the cyanobacterium *Anabaena* sp. Chaurasia et al. (Chaurasia et al., 2013) demonstrated removal of 10 ppm lindane within 6–10 days by overexpression of the *linA2* transgene. The cyanobacterium *S. elongatus* has also been investigated for remediation of chemical pollutants. Han et al. (Han et al., 2020) demonstrated that wild type *S. elongatus* was able to remove 99.5% of malachite green, a carcinogenic dye. However, they noted that the dye was not degraded or chemically modified. Then they heterologous expressed the triphenylmethane reductase gene *katmr*, which demonstrated 99.8% degradation.

### 4.4 Genetically engineered microalgae for plastic degradation

PETase expression in microalgae has been done to a rudimentary scale by Kim et al. (Kim et al., 2020) and Moog et al. (Moog et al., 2019) in *C. reinhardtii* and *P. tricornutum* respectively. Kim et al. utilised the Sh-Ble-2A fusion expression system to integrate the wild type PETase gene, and chemical and morphological changes in the PETase film were detected after 4 weeks. The slow reaction time was likely a factor of PETase activity being blocked by intracellular proteins and compounds, as the enzyme was not secreted in this study. Similarly, the reaction rate of the wild type PETase is significantly lower than mutant proteins. On the other hand, Moog et al. utilised the *Is*PETase^R280A^ mutant gene, combined with an endogenous alkaline phosphatase secretion tag. The activity of the enzyme when secreted was confirmed by scanning electron microscopy of PET plastic after incubation in the culture medium, demonstrating that enzymatic functionality is maintained.

## 5 Research gaps and future directions

This review has clearly demonstrated the potential that exists for genetically engineered microalgae bioremediation systems, yet research advances have been limited by the lack of a consistent and versatile transgenic expression system that could accelerate the rate of mutant strain generation. While some rapid expression kits have been developed for specific model species, such as *C. reinhardtii*, most algal species are yet to see use of such toolkits. To accelerate the research progress in this field, it is essential that the genetic toolkits are expanded to other algal species. Toolkits should be developed following the MoClo design philosophy, and parts should be compatible between toolkits to allow for rapid development of this research.

Genetic engineering can also be used to create new pollutant targets entirely, using transgenic strains. One possible application of this is the treatment of waste PET plastic by green microalgae. This has currently only been tested in chassis strains *C. reinhardtii* (Kim et al., 2020) and *P. tricornitum* (Moog et al., 2019) but has potential to be expanded to microalgae more suited for growth in PET contaminated water sites.

Regarding the use of microalgae for salinity removal, increased cellular growth will predominantly control the rate of inorganics uptake, such as bicarbonates, but there is potential for investigating the genes involved in bicarbonate transport and anhydrase activity, such as carbonic anhydrase and ion influx proteins. Similarly, there is potential for improvement in the structure of the protein shell, potentially increasing retainment of CO_2_ in the carboxysome. However, the most promising method to increase salinity uptake rates is improvement of the bioadsorption capability, through manipulation of the cell surface groups.

There are many routes for synthetic biology-based approaches to improve heavy metal remediation in microalgae. Chelatin synthase genes, membrane bound transporter and receptor proteins, and transcriptional regulators all offer meaningful targets for strain improvement, yet expanding synthetic biology tools to more under-used algal strains could provide a larger benefit overall.

While many microalgal species have demonstrated bioremediation capability for pesticides, the wide range of target chemicals and complex mechanisms of action means that any bioremediation strategy must be tailor-made for the specific contamination issue. However, the use of genetic engineering to incorporate transgenic metabolic pathways and functional groups may allow for a wider range of chemicals to be targeted by a specific organism. Additionally, genetic engineering could play a role in increasing species tolerance, allowing for more significant uptake of inorganic and organic contaminants.

Among studies of genetically engineered microalgae, there has been a significant lack of large-scale cultivation. Current large-scale microalgal systems typically involve open raceway systems or ponds, which are inadequate for genetically engineered microalgae from a biosecurity standpoint. Therefore, large-scale cultivation could increase the capital and operating costs due to the need of a contained systems and would require in-depth and effective risk assessment of these (Beacham et al., 2017). Genetically modified microalgae must therefore balance the increased cost of contained cultivation with significant improvements in pollutant uptake rates to be viable. So far, research has been limited in this regard.

## 6 Conclusion

Microalgae have great potential for developing sustainable technologies to remove inorganic and organic pollutants from the environment. This potential is attributed to various mechanisms such as biosorption, bioaccumulation, and biodegradation, involving different functional groups, proteins, and peptides responsible for pollutant-binding and detoxification.

Synthetic biology offers a promising approach to enhance these mechanisms by manipulating gene expression, introducing DNA into cells, and generating mutants with altered phenotypes, thereby expanding the range of pollutant targets and increasing removal and transformation rates. However, the application of universal synthetic biology tools for microalgae needs further expansion to fully realize this potential.

The most studied species for bioremediation among microalgae and cyanobacteria include *C. vulgaris*, *P. tricornutum*, *N. oceanica*, *A. platensis*, and *C. reinhardtii*, with the latter being the most widely genetically engineered and studied. Current genetic tools applied to microalgae include plasmid-based systems and gene integration techniques such as conjugation, electroporation, bombardment, episomal DNA expression, transposons, and Agrobacterium tumefaciens. Furthermore, precise genome engineering, gene inactivation/knockout, and gene insertion (knock-in) have been achieved in some species using CRISPR/Cas9, transposons, and episomal DNA expression. Future research should focus on developing rapid expression kits and expanding universal cloning toolkits to adapt genetic engineering tools to a wider range of microalgae species.
