# Supplementary material for: A synthetic biology approach for the treatment of pollutants with microalgae
Source: Front Bioeng Biotechnol. 2024 Apr 5;12:1379301. doi: 10.3389/fbioe.2024.1379301 (PMC11032018; doi:10.3389/fbioe.2024.1379301)
Supplement: Supplementary file 1 [file Table1.DOCX]

**Supplementary Information**

**Table S1:** Significant pollutant treatment research using microalgae.

| **Category** | **Species** | **Primary remediation applications** | **Results** | **Type** | **References** |
| --- | --- | --- | --- | --- | --- |
| Cyano-bacteria | Anabaena sp. | Lindane (γ-HCH) pesticide | >98% of 10ppm lindane degraded in 6-10 days | GE | [1] |
|  |  | Zinc | 2.97 mg zinc per g DCW | Wild type | [2] |
|  | Azolla-anabaena plant-microbe symbiont | U, Fe, Mn, Cu, Zn, Pb, Cd | 87.6, 99.1, 98.8, 88.2, 91, 78.3, 77.5% reduction respectively | Wild type (symbiont with plant) | [3] |
|  | Arthrospira platensis | Textile effluent (Ismate violet dye) | 75.7 and 61.1% reduction in dry biomass and lipid free biomass respectively | Wild type | [4] |
|  |  | Erbium (III) | >65% removal of 100mg/L Er (III) | Wild type | [5] |
|  |  | Cu, Ni, Al | 95% metals reduced overall in 140 minutes | Wild type | [6] |
|  | Arthrospira maxima | Sulfate | 3000mg/L reduced by 73% | Wild type | [7] |
|  | Synechococcus elongatus | Malachite green dye | 99.5% of 100mg/L dye removed | GE | [8] |
|  |  | Salinity (NaCl) | Growth rate increased by 57.7% in 0.4M NaCl | GE | [9] |
|  | Synochococcus-pseudomonas co-culture | 2,4-dinitrotoluene | 136g/L removed in 15 days | GE | [10] |
| Green algae | Chlorella vulgaris | Ni, Zn, Cd, Cu | 50, 79, 85 and 80% biosorption efficiency respectively | Wild type | [11] |
|  |  | Cu, Ni, Al | 87% metals reduced overall in 140 minutes | Wild type | [6] |
|  |  | Methylene blue dye | 99.7% methylene blue decolorisation | Wild type | [12] |
|  |  | Distillery wastewater | TN, TP and COD removal efficiency of 80, 94 and 72.24% | Wild type | [13] |
|  |  | CO_2_ | 1.2-fold increase in photosynthetic ability | GE | [14] |
|  | Chylamydomonas reinhardtii | Phenol | ~900µmol/L phenol removed in 8 days | Wildtype | [15] |
|  |  | Cyanide | Up to 150mg/L potassium cyanide remediated | GE | [16] |
|  |  | Arsenic (III) | 95.2% of 25mg/L arsenic removed | Wild type | [17] |
|  |  | Manganese and cadmium | 2.3-fold increase in Mn uptake & cadmium tolerance increase | GE | [18] |
|  |  | Polyethylene terephthalate (PET) | 35.17% degradation rate of PET after 4 weeks | GE | [19] |
|  |  | Cadmium and manganese | 2-3-fold increase in Mn uptake | GE | [18] |
|  |  | Cadmium and Zinc | Two-threefold increase in uptake of Cd and Zn | GE | [20] |
|  |  | Inorganic carbon/CO2 | 1.3-fold increase in inorganic carbon uptake | GE | [21] |
|  |  | Penoxsulam pesticide | 93.6 and 54% removal compared to 52 and 21% removal for wildtype | GE | [22] |
|  | Chylamydomonas sp. (ChlSP) | Uranium | 6.34mg U per g DCW | Artificial evolution | [23] |
|  | Dunaliella sp. | Chromium (VI) | 66.4% biosorption efficiency | Wildtype | [24] |
|  |  | Desalination (chloride, sodium, bicarbonate) | 1500mg/L reduction of sodium chloride, 8mg/L reduction of bicarbonate | Wild type | [25] |
|  | Haematococcus sp. | Crude oil | 32% reduction in hydrocarbon concentration | Wild type | [26] |
|  |  | Wastewater | 39.3% and 90.9% reduction in nitrogen and phosphorus respectively | Wild type | [27] |
|  | Nannochloropsis oculata | Lead | Up to 55% lead removal after 7 days | Wild type | [28] |
|  | Nannochloropsis oceanica | Dairy wastewater | 57, 100 and 95% reduction in lactose, nitrate and phosphate after 30 days | Wild type | [29] |
|  |  | Municipal and agricultutal wastewater | 99% uptake of nitrogen and phosphate | Wild type | [30] |
|  | Scenedesmus sp. | Chromium (VI) | 99.4% removal of 10ppm Cr (VI) in heterotrophic conditions | Wild type | [31] |
|  |  | Orange-2RL Azo dye | 98.14% removal at 20ppm azo dye | Wild type | [32] |
|  | Rhinomonas reticulata | Propylbenzenes | 72% and 56% of n-PBZ and i-PBZ degraded after 7 days | Wild type | [33] |
|  | Uronema africanum | Low density Polyethylene (LDPE) | Fully colonised plastic sheet after 30 days incubation, decay detected by SEM | Wild type | [34] |
| Diatoms | Phaeodactylum tricornitum | Oilfield produced water | 92, 76, 85, 72 and 56% removal of NO3-, PO4.3-, Fe, F and Mg respectively | Wild type | [35] |
|  |  | Polyethylene terephthalate (PET) | PET degradation observed, reaction products detected by HPLC (not quantified) | GE | [36] |
|  |  | Sulfadimethoxine (SDM) antibiotic | 100% removal of 0.5 mg/L SDM | Wild type | [37] |

1. Chaurasia, A.K., T.K. Adhya, and S.K. Apte, *Engineering bacteria for bioremediation of persistent organochlorine pesticide lindane (γ-hexachlorocyclohexane).* Bioresour. Technol., 2013. **149**: p. 439-445.

2. Chakraborty, S., et al., *Deciphering the mechanisms of zinc tolerance in the cyanobacterium Anabaena sphaerica and its zinc bioremediation potential.* Environ. Sci. Pollut. Res. Int., 2023. **30**(4): p. 9591-9608.

3. Xinwei, H., et al., *Bioremediation of effluent from a uranium mill tailings repository in South China by Azolla–Anabaena.* J. Radioanal. Nucl. Chem., 2018. **317**(2).

4. Alprol, A.E., et al., *Potential applications of arthrospira platensis lipid-free biomass in bioremediation of organic dye from industrial textile effluents and its influence on marine rotifer (Brachionus plicatilis).* Materials, 2021. **14**(16): p. 4446.

5. Yushin, N., et al., *Application of Cyanobacteria Arthospira platensis for Bioremediation of Erbium-Contaminated Wastewater.* Materials, 2022. **15**(17): p. 6101.

6. Almomani, F. and R.R. Bhosale, *Bio-sorption of toxic metals from industrial wastewater by algae strains Spirulina platensis and Chlorella vulgaris: Application of isotherm, kinetic models and process optimization.* Sci. Total Environ., 2021. **755**: p. 142654.

7. Blanco-Vieites, M., et al., *Removal of heavy metals and hydrocarbons by microalgae from wastewater in the steel industry.* Algal Res., 2022. **64**: p. 102700.

8. Han, S., et al., *Bioremediation of malachite green by cyanobacterium Synechococcus elongatus PCC 7942 engineered with a triphenylmethane reductase gene.* Appl. Microbiol. Biotechnol., 2020. **104**(7): p. 3193-3204.

9. Cui, J., et al., *Improved Salt Tolerance and Metabolomics Analysis of Synechococcus elongatus UTEX 2973 by Overexpressing Mrp Antiporters.* Front. Bioeng. Biotechnol., 2020. **8**: p. 500-500.

10. Fedeson, D.T., et al., *Biotransformation of 2,4‐dinitrotoluene in a phototrophic co‐culture of engineered Synechococcus elongatus and Pseudomonas putida.* Microb. Biotechnol., 2020. **13**(4): p. 997-1011.

11. Piccini, M., et al., *A synergistic use of microalgae and macroalgae for heavy metal bioremediation and bioenergy production through hydrothermal liquefaction.* Sustain. Energy Fuels., 2019. **3**(1): p. 292-301.

12. Fazal, T., et al., *Integrating bioremediation of textile wastewater with biodiesel production using microalgae (Chlorella vulgaris).* Chemosphere, 2021. **281**: p. 130758-130758.

13. Li, F., et al., *Cultivation of Chlorella vulgaris in Membrane-Treated Industrial Distillery Wastewater: Growth and Wastewater Treatment.* Front. Environ. Sci., 2021. **9**.

14. Yang, B., et al., *Genetic engineering of the Calvin cycle toward enhanced photosynthetic CO2 fixation in microalgae.* Biotechnol. Biofuels, 2017. **10**(1): p. 229-229.

15. Nazos, T.T., et al., *Biodegradation of phenol by Chlamydomonas reinhardtii.* Photosynth. Res., 2020. **144**(3): p. 383-395.

16. Sobieh, S.S., et al., *Heterologous Expression of Cyanobacterial Cyanase Gene (CYN) in Microalga Chlamydomonas reinhardtii for Bioremediation of Cyanide Pollution.* Biology (Basel), 2022. **11**(10): p. 1420.

17. Mohamed, M.S., et al., *Adsorptive recovery of arsenic (III) ions from aqueous solutions using dried Chlamydomonas sp.* Heliyon, 2022. **8**(12): p. e12398-e12398.

18. Ibuot, A., et al., *Metal bioremediation by CrMTP4 over-expressing Chlamydomonas reinhardtii in comparison to natural wastewater-tolerant microalgae strains.* Algal Res. , 2017. **24**: p. 89-96.

19. Kim, J.W., et al., *Functional expression of polyethylene terephthalate-degrading enzyme (PETase) in green microalgae.* Microb. Cell Fact., 2020. **19**(1): p. 97-97.

20. Ibuot, A., et al., *Increased metal tolerance and bioaccumulation of zinc and cadmium in Chlamydomonas reinhardtii expressing a AtHMA4 C‐terminal domain protein.* Biotechnol. Bioeng., 2020. **117**(10): p. 2996-3005.

21. Ohnishi, N., et al., *Expression of a Low CO₂-Inducible Protein, LCI1, Increases Inorganic Carbon Uptake in the Green Alga Chlamydomonas reinhardtii.* Plant Cell, 2010. **22**(9): p. 3105-3117.

22. Ismaiel, M., Y. El-Ayouty, and A. Al-Badwy, *Biosorption Analysis and Penoxsulam Herbicide Removal Efficiency by Transgenic Chlamydomonas reinhardtii Overexpression the Cyanobacterial Enzyme Glutathione-s- transferase.* Jordan J. Biol. Sci., 2019. **12**(5): p. 617-624.

23. Baselga-Cervera, B., et al., *Improvement of the uranium sequestration ability of a Chlamydomonas sp. (ChlSP strain) isolated from extreme uranium mine tailings through selection for potential bioremediation application.* Front. Microbiol., 2018. **9**: p. 523-523.

24. Vidyalaxmi, G. Kaushik, and K. Raza, *Potential of novel Dunaliella salina from sambhar salt lake, India, for bioremediation of hexavalent chromium from aqueous effluents: An optimized green approach.* Ecotoxicol. Environ. Saf., 2019. **180**: p. 430-438.

25. Moayedi, A., et al., *Investigation of bio-desalination potential algae and their effect on water quality.* Desalination Water Treat., 2021. **212**: p. 78-86.

26. Radice, R.P., et al., *Bioremediation of Crude Oil by Haematococcus Pluvialis: A Preliminary Study.* Processes, 2022. **10**(12): p. 2472.

27. Kashem, A.H.M., et al., *Microalgal bioremediation of brackish aquaculture wastewater.* Sci. Total Environ., 2023. **873**: p. 162384.

28. Waluyo, L., et al. *Potential bioremediation of lead (Pb) using marine microalgae Nannochloropsis oculata*. in *AIP Conference* 2020.

29. Li, Y., et al., *Mechanism of lactose assimilation in microalgae for the bioremediation of dairy processing side-streams and co-production of valuable food products.* J. Appl. Phycol., 2023.

30. Silkina, A., et al., *Large-scale waste bio-remediation using microalgae cultivation as a platform.* Energies (Basel), 2019. **12**(14): p. 2772.

31. Kafil, M., et al., *Utilization of the microalga Scenedesmus quadricauda for hexavalent chromium bioremediation and biodiesel production.* Bioresour. Technol., 2022. **346**: p. 126665.

32. Hamouda, R.A., N.E.A. El‑Naggar, and G.W. Abou-El-Souod, *Simultaneous bioremediation of Disperse orange-2RL Azo dye and fatty acids production by Scenedesmus obliquus cultured under mixotrophic and heterotrophic conditions.* Sci. Rep., 2022. **12**(1): p. 20768.

33. Du, S., et al., *Bioremediation of propylbenzenes by a novel marine microalga Rhinomonas reticulata S6A isolated from Daya Bay: acute toxicity, growth kinetics and biodegradation performance.* Front. Mar. Sci., 2023. **10**.

34. Sanniyasi, E., et al., *Biodegradation of low-density polyethylene (LDPE) sheet by microalga, Uronema africanum Borge.* Sci. Rep., 2021. **11**(1): p. 17233.

35. Gillard, J.T.F., et al., *Potential for biomass production and remediation by cultivation of the marine model diatom phaeodactylum tricornutum in oil field produced wastewater media.* Water (Basel), 2021. **13**(19): p. 2700.

36. Moog, D., et al., *Using a marine microalga as a chassis for polyethylene terephthalate (PET) degradation.* Microb. Cell. Fact., 2019. **18**(1): p. 171-171.

37. Li, B., et al., *Metabolic Mechanism of Sulfadimethoxine Biodegradation by Chlorella sp. L38 and Phaeodactylum tricornutum MASCC-0025.* Front. Microbiol., 2022. **13**: p. 840562-840562.
